# Interpretable detection of left ventricular hypertrophy using commercial ECG features and machine learning: a study based on the PTB-XL+ dataset

**DOI:** 10.3389/fcvm.2026.1825829

**Published:** 2026-06-04

**Authors:** Qibao Zhou, Xiao Luo, Kaihui Du

**Affiliations:** Department of Cardiovascular Medicine, Jiujiang City Key Laboratory of Cell Therapy, The First Hospital of Jiujiang City, Jiujiang, China

**Keywords:** electrocardiography, interpretable artificial intelligence, left ventricular hypertrophy, machine learning, PTB-XL+, SHAP

## Abstract

**Introduction and objectives:**

Left ventricular hypertrophy (LVH) predicts cardiovascular morbidity and mortality. ECG is the most accessible LVH screening tool, but conventional voltage criteria have low sensitivity. Deep learning improves detection but lacks interpretability. This study aims to evaluate the utility of PTB-XL+ pre-extracted commercial ECG features for interpretable LVH detection.

**Methods:**

We retrospectively analyzed 11,692 PTB-XL+ ECG records (2,258 LVH, 9,434 normal). Three feature sets (GE 12SL:782, UNIG:702, ECGDeli:529) and four classifiers (XGBoost, LightGBM, Random Forest, Logistic Regression) were evaluated via five-fold stratified cross-validation. SHAP analysis provided feature importance interpretations.

**Results:**

XGBoost performed best (AUC: 12SL = 0.9859, UNIG = 0.9853, ECGDeli = 0.9828). Commercial features marginally outperformed open-source ECGDeli. SHAP identified key features (V5–V6T-wave changes, precordial R-wave amplitude, V1 QRS area) and age as an important non-ECG predictor.

**Conclusion:**

Interpretable machine learning (ML) with commercial ECG features achieves excellent LVH detection with clinically meaningful attributions, bridging high performance and interpretability for clinical integration.

## 1 Introduction

Left ventricular hypertrophy (LVH) is a pathological increase in left ventricular mass that serves as an independent risk factor for heart failure, stroke, sudden cardiac death, and overall cardiovascular mortality (1, 2). Early detection and management of LVH can significantly reduce the burden of adverse cardiovascular events. While echocardiography and cardiac magnetic resonance imaging remain the gold standards for LVH diagnosis, the 12-lead electrocardiogram (ECG) is the most widely available, cost-effective, and frequently performed cardiovascular diagnostic test in clinical practice (3).

Traditional ECG criteria for LVH, such as the Sokolow–Lyon voltage criteria and the Cornell product, are limited by modest sensitivity (typically 20%–50%) despite high specificity (4, 5). This diagnostic gap has motivated extensive research into computational approaches for improving ECG-based LVH detection. In recent years, deep learning models applied directly to raw ECG waveforms have demonstrated remarkable performance, with several studies reporting AUC values exceeding 0.90 (6, 7). However, the black-box nature of these models poses significant barriers to clinical adoption, as clinicians require transparent reasoning processes to trust and integrate automated diagnostic tools into patient care (8).

The PTB-XL dataset, one of the largest publicly available clinical ECG databases comprising 21,799 records from 18,885 patients, has become a benchmark for ECG classification research (9). Its extension, PTB-XL+, enriches the original dataset with pre-computed features from three established ECG analysis programs: the GE 12SL algorithm, the University of Glasgow (UNIG) ECG analysis program, and the open-source ECGDeli toolkit (10). These commercial-grade features encode morphological, temporal, and amplitude measurements that mirror the parameters cardiologists assess during manual ECG interpretation. Despite the availability of these expertly engineered features, their potential for interpretable LVH detection using traditional machine learning methods remains largely unexplored.

Furthermore, existing benchmarks on the PTB-XL dataset have consistently identified LVH as one of the most challenging diagnostic categories, with substantially lower classification performance compared to other ECG abnormalities (9, 10). This underperformance highlights the need for focused investigations into optimized detection strategies for this clinically important condition. The wider clinical landscape of LVH detection, encompassing both imaging-based and ECG-based approaches, further motivates the development of interpretable and accessible ECG analysis tools that can serve as a complementary screening layer to confirmatory imaging.

## 2 Objectives

**Figure 1:**
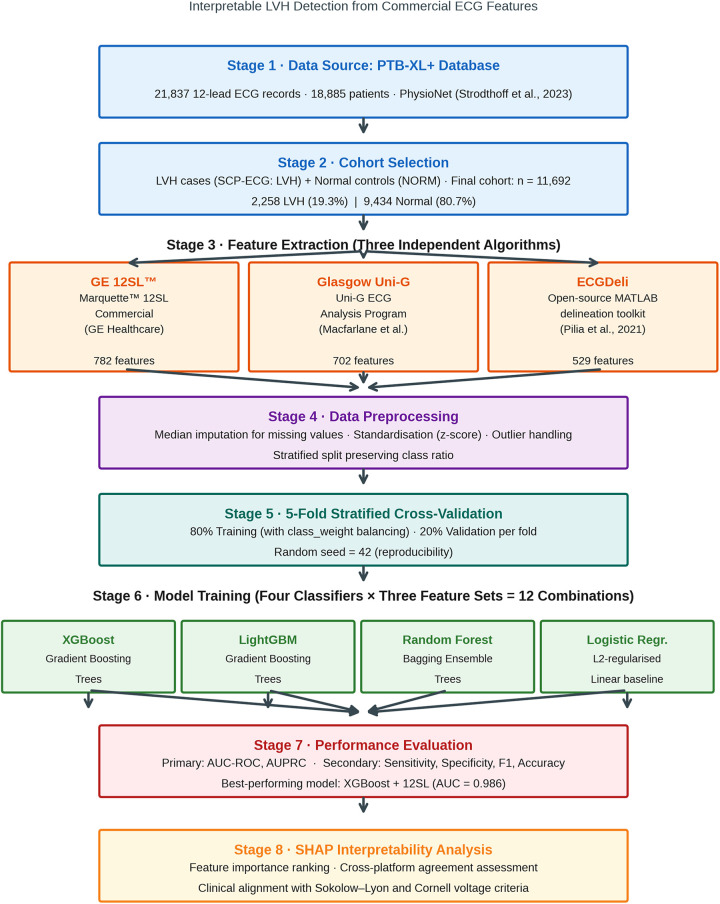
System architecture of the proposed framework. The pipeline comprises eight stages: (1) data sourcing from PTB-XL+; (2) cohort selection (*n* = 11,692; 2,258 LVH and 9,434 Normal); (3) parallel feature extraction by three independent ECG analysis programs (GE 12SL, Glasgow Uni-G, ECGDeli); (4) data preprocessing including median imputation and z-score standardisation (for Logistic Regression); (5) five-fold stratified cross-validation; (6) training of four classifiers (XGBoost, LightGBM, Random Forest, Logistic Regression) on each feature set, yielding 12 model–feature combinations; (7) performance evaluation using AUC-ROC, AUPRC, sensitivity, specificity, and F1; and (8) SHAP-based interpretability analysis. [insert architecture diagram image here.].

In this study, we systematically evaluate the utility of pre-extracted ECG features from three analysis programs (12SL, UNIG, and ECGDeli) for LVH detection using four machine learning classifiers. We employ SHAP (SHapley Additive exPlanations) analysis to provide comprehensive interpretability at both global and local levels, enabling clinicians to understand which ECG parameters drive model predictions. Our approach bridges the gap between high-performance automated detection and the clinical interpretability required for responsible deployment in healthcare settings. The principal contributions of this study are threefold. First, we present the first systematic evaluation of three commercial-grade ECG feature sets in PTB-XL+ (12SL, Uni-G, ECGDeli) for interpretable LVH detection using traditional machine learning. Second, our cross-platform comparison of feature importance, enabled by SHAP analysis applied across three independently developed algorithms, reveals both shared and platform-specific electrocardiographic signatures of LVH that, to our knowledge, have not been systematically reported. Third, we demonstrate that interpretable tree-based models with engineered features can achieve performance on par with deep learning approaches reported in the recent literature, while preserving the clinical transparency that is essential for safe deployment. The overall system architecture and workflow of the proposed framework are schematically illustrated in Figure 1.

## 3 Materials and methods

### 3.1 Dataset

This study utilized the PTB-XL+ dataset, an enriched version of the PTB-XL electrocardiography dataset hosted on PhysioNet (9, 10). The PTB-XL dataset contains 21,799 clinical 12-lead ECG records from 18,885 patients, recorded at a sampling rate of 500 Hz over 10-second durations at the Schiller AG facilities between 1989 and 1996. Each record is annotated with up to three diagnostic statements conforming to the SCP-ECG standard, with likelihood scores assigned by cardiologists. Records were originally acquired with multiple Schiller AG devices (CS-100, AT-60/100, AT-101); PTB-XL provides all signals resampled to 500 Hz. Diagnostic labels were generated by two independent cardiologists and reconciled, following the SCP-ECG standard. The dataset spans patient ages of 0–95 years with balanced sex representation overall.

For this study, we identified ECG records with LVH (hypertrophy superclass, *n* = 2,258) and normal (NORM superclass, *n* = 9,434) diagnoses, using a likelihood threshold of 50% for diagnostic label assignment. Records with co-occurring LVH and NORM labels were classified as LVH. The final study cohort comprised 11,692 ECG records. Implausible demographic values were recoded as missing and handled through the standard imputation pipeline described below.

### 3.2 ECG feature extraction

PTB-XL+ provides pre-computed features from three ECG analysis programs, each implementing distinct algorithms for waveform delineation and parameter extraction:

**GE 12SL Algorithm:** A proprietary commercial ECG analysis program developed by GE Healthcare, widely deployed in clinical ECG machines worldwide. It extracted 782 features encompassing P-wave, QRS complex, ST-segment, and T-wave measurements across all 12 leads, including amplitude, duration, area, and morphology parameters (see GE Healthcare Marquette 12SL Physician's Guide); the deposited 12SL feature table in PTB-XL+ comprises 782 columns, as determined by direct inspection of the dataset (10).
**University of Glasgow (UNIG) Algorithm:** A well-validated academic ECG interpretation program from the University of Glasgow, recognized for its diagnostic accuracy in large-scale clinical evaluations. It provided 702 features with comprehensive morphological descriptors including vectorcardiographic (VCG) parameters and specialized waveform measurements [see also Macfarlane et al. (18)]; the deposited Uni-G feature table in PTB-XL+ contains 702 columns (10).
**ECGDeli:** An open-source ECG delineation toolkit that provides 529 features focusing on standard amplitude, duration, and interval measurements. While less comprehensive than the commercial alternatives, it represents a freely available and reproducible analysis pipeline [see Pilia et al. (19)] (10).

### 3.3 Data preprocessing

For each feature source, the following preprocessing steps were applied: (1) features with greater than 50% missing values were excluded; (2) constant features (zero variance) were removed; (3) remaining missing values were imputed using median imputation; and (4) demographic variables (age, sex, height, and weight) were appended to the feature matrices. After preprocessing, the feature dimensions were 782 for 12SL, 702 for UNIG, and 529 for ECGDeli. For Logistic Regression, features were standardized using z-score normalization. Tree-based models (XGBoost, LightGBM, and Random Forest) operated on unscaled features.

### 3.4 Machine learning models

Four machine learning classifiers were evaluated to provide a comprehensive comparison across different algorithmic paradigms:

**XGBoost:** Extreme Gradient Boosting was configured with 300 estimators, maximum depth of 6, learning rate of 0.1, and scale_pos_weight adjusted for class imbalance. **LightGBM:** Light Gradient Boosting Machine used identical hyperparameters (300 estimators, maximum depth 6, learning rate 0.1) with class weight adjustment. **Random Forest:** An ensemble of 300 decision trees with maximum depth of 15 and balanced class weights. **Logistic Regression:** L2-regularized logistic regression with balanced class weights and a maximum of 1,000 iterations. Class imbalance was addressed at three levels. First, all classifiers were trained with class-weight balancing (scale_pos_weight for XGBoost/LightGBM, class_weight='balanced' for Random Forest and Logistic Regression). Second, the cross-validation folds were stratified to preserve the LVH:Normal ratio of approximately 1:4.2 in each fold. Third, primary performance was reported using AUC-ROC and AUPRC, both of which are insensitive to prior class probability, rather than raw accuracy.

Model performance was evaluated using five-fold stratified cross-validation to ensure representative class distribution in each fold.Evaluation metrics included the area under the receiver-operating-characteristic curve (AUC-ROC), the area under the precision-recall curve (AUPRC), F1 score, accuracy, sensitivity, and specificity. AUC-ROC was used as the primary metric. Sensitivity = TP/(TP + FN); Specificity = TN/(TN + FP); F1 = 2 × Precision × Recall/(Precision + Recall). All metrics were computed on each held-out validation fold and averaged across the five folds, reported as mean ± standard deviation. The default classification threshold of 0.5 was used; as a sensitivity analysis, we additionally identified the optimal threshold via the Youden J statistic on each fold and confirmed that classification performance was robust to threshold variation. All experiments used a fixed random seed (set to 42, an arbitrary integer commonly adopted in the data-science community as a reproducibility convention; results were stable across alternative seed choices in pilot tests) for reproducibility.

### 3.5 SHAP interpretability analysis

To provide mechanistic insights into model predictions, we employed SHAP (SHapley Additive exPlanations) analysis based on game-theoretic Shapley values (11). For each feature source, the best-performing XGBoost model was retrained on the complete dataset, and TreeExplainer was used to compute exact SHAP values for all samples. Global feature importance was quantified by the mean absolute SHAP value across all samples for each feature. SHAP summary (beeswarm) plots visualized the distribution of feature contributions, while dependence plots revealed the relationship between individual feature values and their SHAP contributions, colored by interaction effects.

### 3.6 Statistical analysis

Continuous variables were presented as mean ± standard deviation or median (interquartile range), as appropriate. Group comparisons were performed using the Mann–Whitney *U* test for continuous variables and the chi-square test for categorical variables. Non-normality was confirmed by the Shapiro–Wilk test. A two-sided *p*-value <0.05 was considered statistically significant. All analyses were performed using Python 3.10 with scikit-learn 1.3, XGBoost 2.0, LightGBM 4.1, and SHAP 0.44.

## 4 Results

### 4.1 Patient characteristics

The study cohort comprised 11,692 ECG records, including 2,258 LVH cases (19.3%) and 9,434 normal controls (80.7%). Baseline demographic characteristics are summarized in Table 1. The LVH group was significantly older than the normal group (66.2 ± 13.7 vs. 52.0 ± 17.2 years, *p* < 0.001) and had a higher proportion of males (58.5% vs. 46.1%, *p* < 0.001). Height and weight data were available for 34.7% and 52.1% of the cohort, respectively, reflecting the retrospective nature of the dataset. The LVH group demonstrated slightly lower height (166.1 ± 10.5 vs. 167.1 ± 10.8 cm, *p* = 0.014) and weight (68.8 ± 17.7 vs. 71.3 ± 15.2 kg, *p* < 0.001) compared to normal controls.

**Table 1:** Baseline characteristics of the study population.

Variable	LVH (*n* = 2,258)	Normal (*n* = 9,434)	*p*-value
Age (years), mean ± SD	66.2 ± 13.7	52.0 ± 17.2	<0.001
Age (years), median (IQR)	68.0 (59.0–76.0)	53.0 (40.0–65.0)	
Male, *n* (%)	1,322 (58.5)	4,347 (46.1)	<0.001
Height (cm), mean ± SD*	166.1 ± 10.5	167.1 ± 10.8	0.014
Weight (kg), mean ± SD^†^	68.8 ± 17.7	71.3 ± 15.2	<0.001

IQR, interquartile range; SD, standard deviation.

* Height data available for 4,058 records (34.7%).

† Weight data available for 6,094 records (52.1%).

### 4.2 Classification performance

**Figure 2:**
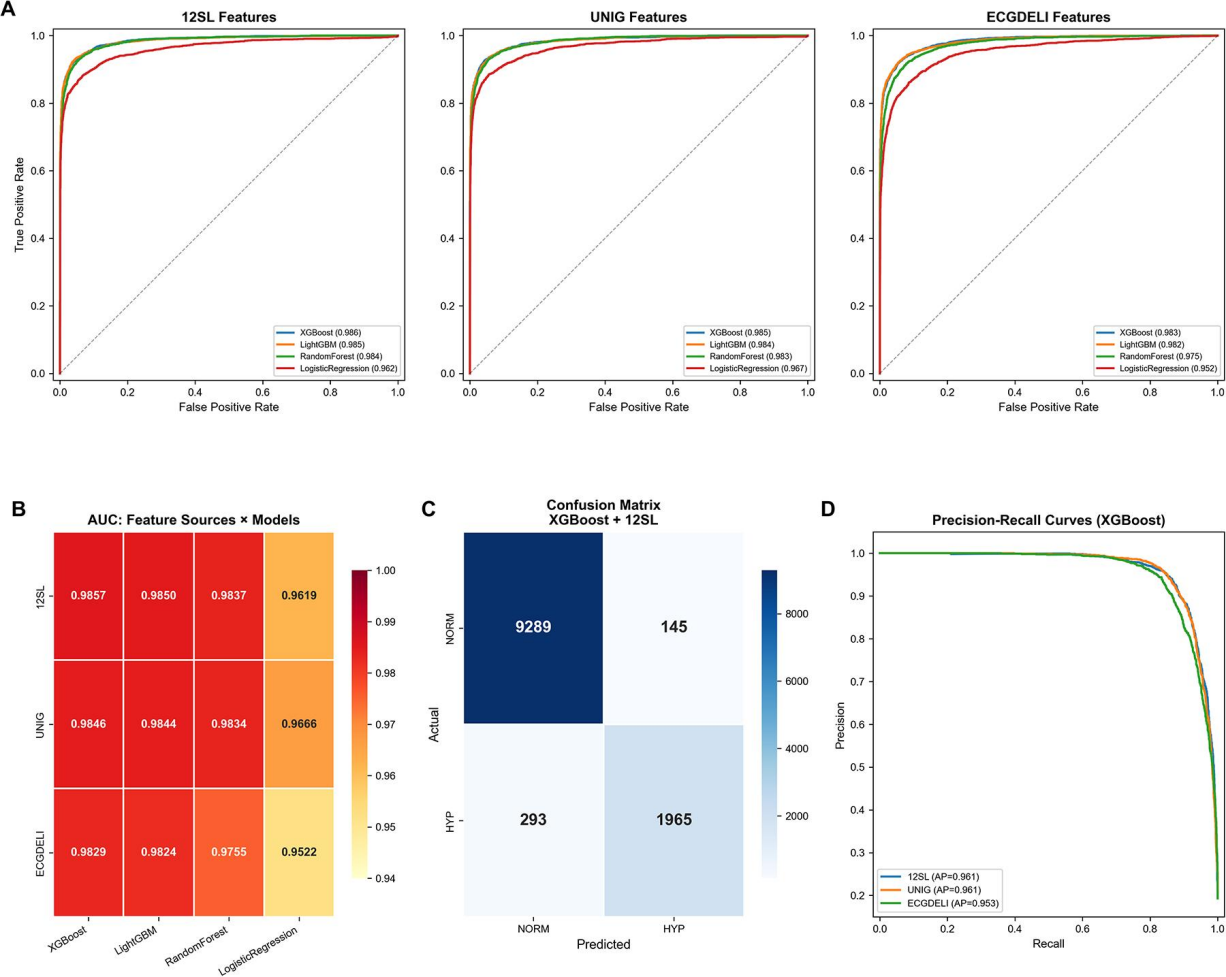
Model performance overview for LVH detection. **(A)** Receiver operating characteristic (ROC) curves for four classifiers across three feature sources (12SL, UNIG, ECGDELI). **(B)** Heatmap of AUC values across feature sources and models. **(C)** Confusion matrix for the best-performing model (XGBoost + 12SL). **(D)** Precision–recall curves comparing feature sources using XGBoost.

The classification performance of all model–feature combinations is presented in Table 2 and visualized in Figure 2. XGBoost consistently achieved the highest AUC across all three feature sources: 0.9859 ± 0.0015 (12SL), 0.9853 ± 0.0016 (UNIG), and 0.9828 ± 0.0017 (ECGDELI). LightGBM performed comparably, followed by Random Forest and Logistic Regression. The gradient boosting methods (XGBoost and LightGBM) demonstrated superior and nearly equivalent performance, while Logistic Regression showed the largest performance gap, particularly with ECGDELI features (AUC = 0.9516 ± 0.0061).

**Table 2:** Classification performance of machine learning models across feature sources (5-fold cross-validation).

Feature source	Model	AUC	F1	Accuracy	Precision	Recall
12SL	XGBoost	0.9859 ± 0.0015	0.9003 ± 0.0051	0.9625	0.9242	0.8778
	LightGBM	0.9854 ± 0.0014	0.8994 ± 0.0025	0.9620	0.9213	0.8787
	Random Forest	0.9827 ± 0.0009	0.8761 ± 0.0076	0.9556	0.9500	0.8131
	Logistic Regression	0.9739 ± 0.0030	0.8492 ± 0.0098	0.9388	0.8102	0.8924
UNIG	XGBoost	0.9853 ± 0.0016	0.9008 ± 0.0038	0.9630	0.9333	0.8707
	LightGBM	0.9847 ± 0.0015	0.8950 ± 0.0027	0.9606	0.9219	0.8698
	Random Forest	0.9825 ± 0.0011	0.8670 ± 0.0042	0.9530	0.9568	0.7927
	Logistic Regression	0.9752 ± 0.0024	0.8422 ± 0.0067	0.9349	0.7924	0.8990
ECGDELI	XGBoost	0.9828 ± 0.0017	0.8842 ± 0.0088	0.9565	0.9095	0.8605
	LightGBM	0.9821 ± 0.0017	0.8840 ± 0.0097	0.9560	0.9009	0.8680
	Random Forest	0.9742 ± 0.0029	0.8267 ± 0.0133	0.9400	0.9343	0.7414
	Logistic Regression	0.9516 ± 0.0061	0.7755 ± 0.0123	0.9041	0.7088	0.8570

AUC values presented as mean ± standard deviation across folds. Precision, recall, and accuracy are mean values across folds.

Among feature sources, 12SL and UNIG exhibited comparable performance across all classifiers (Figures 2A,B), while ECGDELI consistently showed marginally lower AUC values. The performance difference between the best (XGBoost + 12SL, AUC = 0.9859) and worst (Logistic Regression + ECGDELI, AUC = 0.9516) configurations was 0.0343, indicating that all feature–model combinations achieved clinically useful discrimination. The AUC heatmap (Figure 2B) further illustrates this pattern, with gradient boosting methods occupying the highest-performing cells across all feature sources. The XGBoost model with 12SL features achieved the highest F1 score (0.9003 ± 0.0051), precision (0.9242), and accuracy (0.9625). Confusion matrix analysis (Figure 2C) for this optimal configuration revealed high specificity (98.3%) with a sensitivity of 87.8%, yielding a positive predictive value of 92.4%. Precision–recall curves (Figure 2D) demonstrated that all three feature sources maintained high precision across a wide range of recall values, with average precision (AP) exceeding 0.95.

### 4.3 Feature importance and interpretability

**Figure 3:**
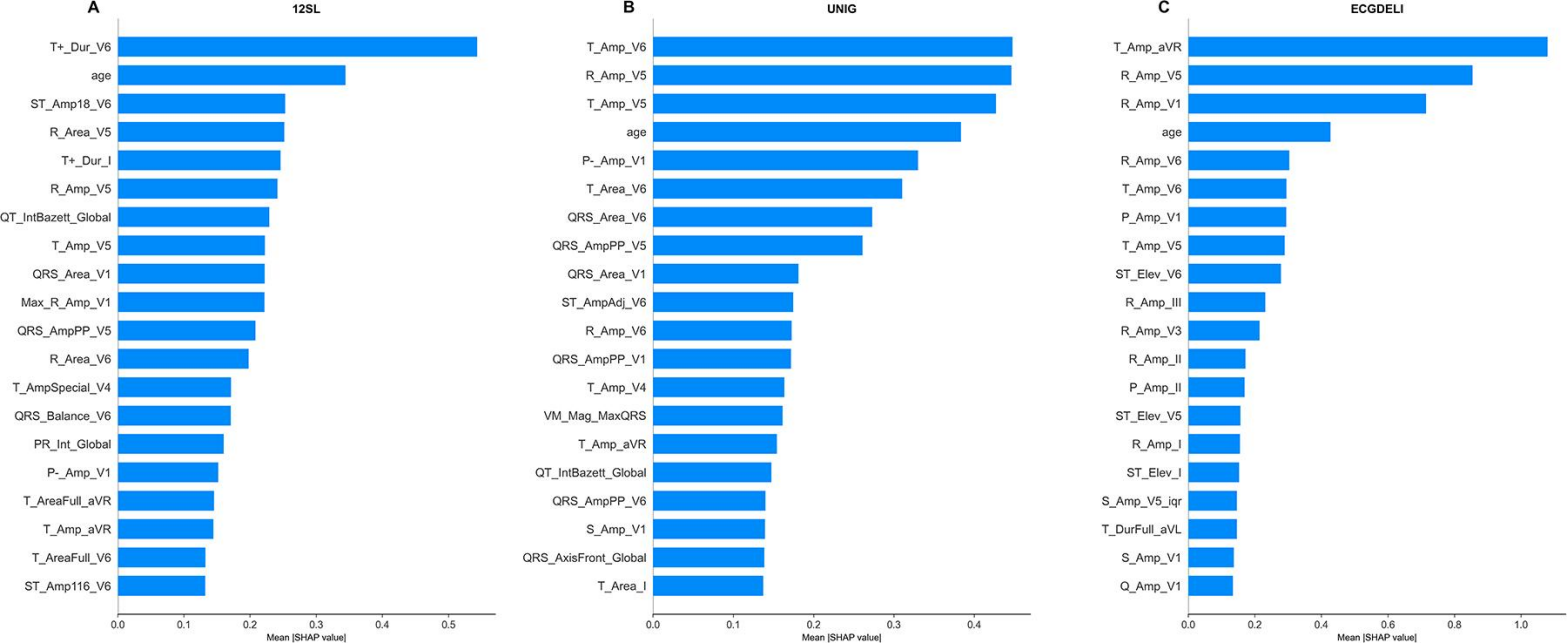
SHAP summary (beeswarm) plots showing the top 20 features for **(A)** 12SL, **(B)** UNIG, and **(C)** ECGDELI feature sets. Each dot represents a sample; color indicates feature value (red = high, blue = low); horizontal position indicates SHAP contribution to the prediction.

**Figure 4:**
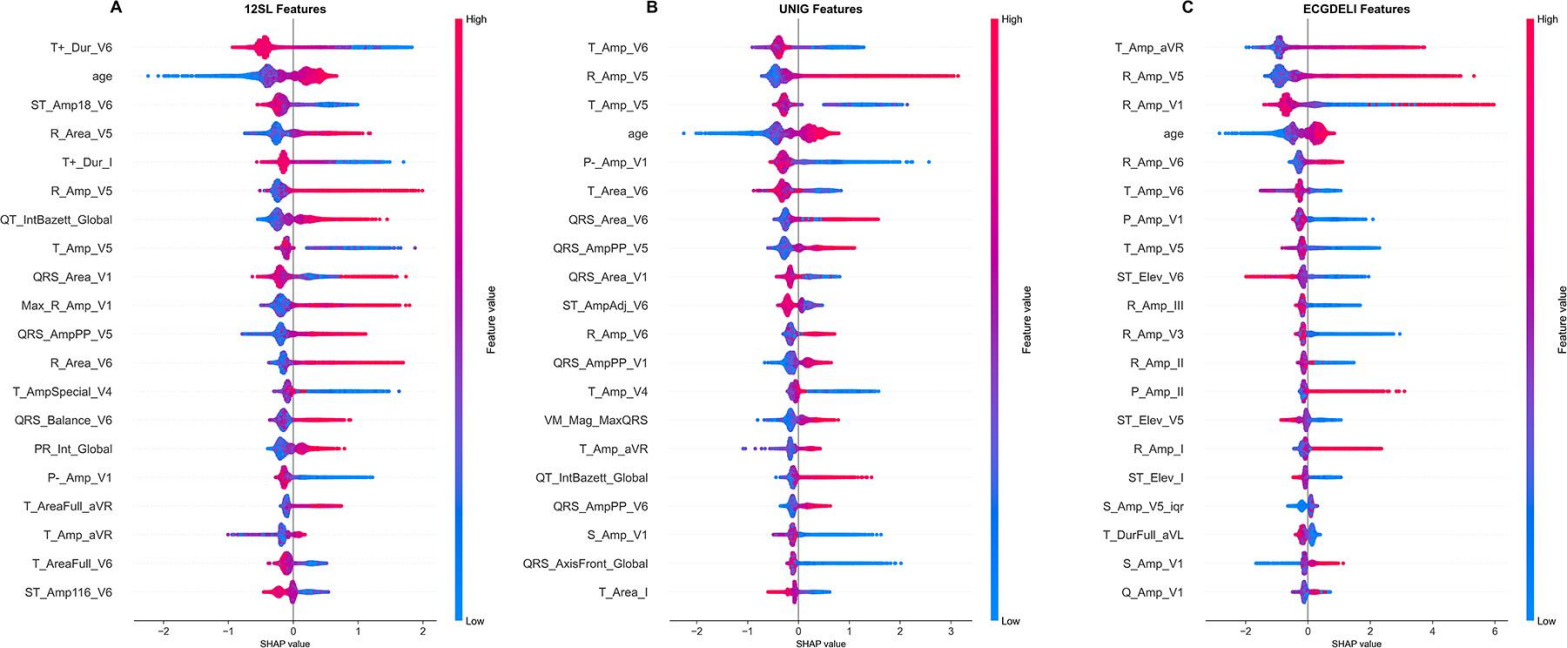
SHAP bar plots of mean absolute SHAP values for the top 20 features across **(A)** 12SL, **(B)** UNIG, and **(C)** ECGDELI feature sets, representing global feature importance rankings.

SHAP analysis revealed distinct yet clinically coherent feature importance patterns across the three feature sources (Figures 3, 4). For the 12SL feature set, T-wave duration in lead V6 (T + _Dur_V6) emerged as the most influential predictor (mean |SHAP| = 0.438), followed by ST-segment deviation in V6 (ST_Amp18_V6, mean |SHAP| = 0.322) and age (0.289). Additional top-ranked features included QRS area in V1 (QRS_Area_V1, 0.209) and R-wave area in V6 (R_Area_V6, 0.202). The dominance of T-wave and ST-segment features in leads V5–V6 directly reflects the repolarization abnormalities characteristic of the LVH strain pattern.

The UNIG feature set exhibited a similar pattern, with T-wave amplitude in V6 (T_Amp_V6) ranking first (mean |SHAP| = 0.383), followed by T-wave area in V6 (T_Area_V6, 0.334) and T-wave amplitude in V5 (T_Amp_V5, 0.333). Age ranked fourth (0.314), and R-wave amplitude in V5 (R_Amp_V5) ranked fifth (0.275). The clustering of T-wave parameters among the top features underscores the central role of repolarization abnormalities in LVH detection. Notably, UNIG features also included vectorcardiographic parameters (VM_Mag_MaxQRS, VM_LenTrans_MaxQRS) among the top 20 features (Figure 3B), suggesting that spatial QRS loop characteristics provide complementary diagnostic information.

The ECGDELI feature set prioritized T-wave amplitude in aVR (T_Amp_aVR) as the top predictor (mean |SHAP| = 0.984), followed by R-wave amplitudes in V5 (R_Amp_V5, 0.584) and V1 (R_Amp_V1, 0.520). R-wave amplitude in V6 ranked fourth (R_Amp_V6, 0.330), and age ranked fifth (0.323). The substantially higher SHAP magnitude of T_Amp_aVR in ECGDELI compared to other features suggests that this feature captures a unique aspect of LVH-related repolarization changes that is not separately quantified in the commercial algorithms.

**Figure 5:**
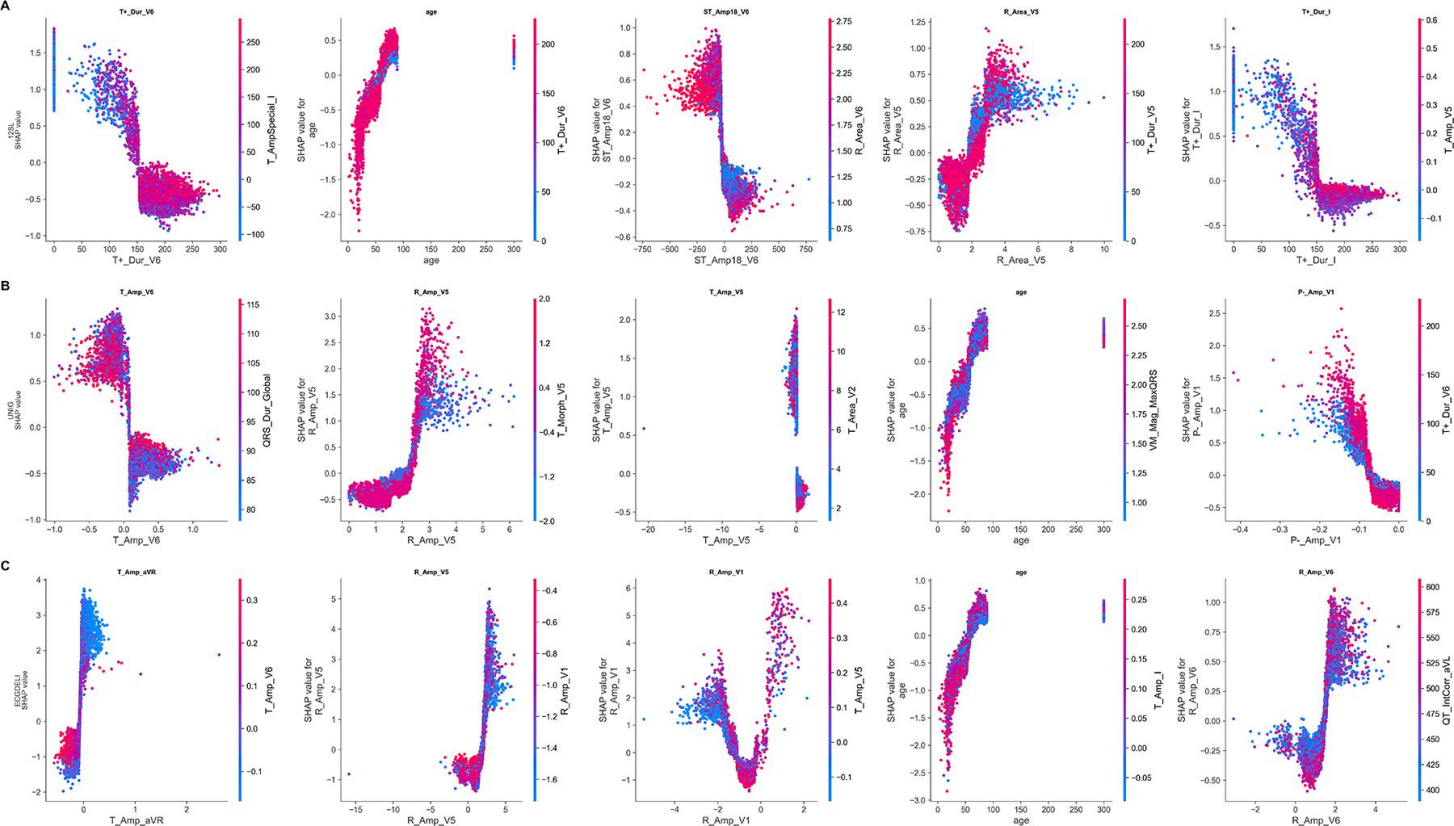
SHAP dependence plots for the top 5 features across three feature sources: **(A)** 12SL, **(B)** UNIG, and **(C)** ECGDELI. Each subplot shows the relationship between a feature value (*x*-axis) and its SHAP contribution (*y*-axis), with color indicating the value of the strongest interacting feature.

Across all three feature sources, age consistently ranked among the top five predictors, reflecting the well-established epidemiological association between advancing age and LVH prevalence. SHAP dependence plots (Figure 5) revealed non-linear relationships between feature values and model predictions, particularly for T-wave measurements where threshold effects were observed, consistent with the clinical distinction between normal repolarization and LVH strain patterns. Interaction effects (indicated by color variations in Figure 5) demonstrated that the significance of individual ECG parameters depends on the overall electrocardiographic context, capturing the multivariate nature of LVH diagnosis.

## 5 Discussion

This study demonstrates that interpretable machine learning models utilizing pre-extracted commercial ECG features can achieve excellent diagnostic performance for LVH detection, with AUC values exceeding 0.98 for gradient boosting methods across all three feature sources. To our knowledge, this is the first systematic evaluation of PTB-XL+ commercial-grade features for LVH-specific detection using interpretable machine learning approaches with comprehensive SHAP analysis. Our findings establish that the combination of expert-engineered ECG features with modern tree-based ensemble methods offers a compelling alternative to end-to-end deep learning for ECG-based LVH screening.

### 5.1 Comparison with deep learning approaches

Our results compare favorably with existing deep learning approaches applied to the PTB-XL dataset. The original PTB-XL benchmark reported macro-averaged AUC values of 0.919–0.936 for the hypertrophy superclass using deep neural networks (including ResNet, Inception, and LSTM architectures) applied to raw ECG signals (9). Strodthoff et al. achieved AUC values of 0.928–0.946 for HYP classification using various deep learning architectures on PTB-XL+ (10). In contrast, our best model (XGBoost + 12SL) achieved an AUC of 0.9859, representing a substantial improvement of approximately 4–6 percentage points over the best reported deep learning results on the same dataset.

This performance advantage likely stems from several factors. First, the focused binary classification task (LVH vs. Normal) simplifies the learning problem compared to multi-label, multi-class classification frameworks used in prior benchmarks. Second, the commercial ECG analysis programs that generate our input features encapsulate decades of domain-specific algorithmic development, effectively providing a highly refined feature engineering pipeline. These programs incorporate expert knowledge about waveform morphology, noise reduction, and lead-specific measurements that raw-signal deep learning models must learn from scratch. Third, gradient boosting methods are particularly well-suited for tabular data with heterogeneous feature types, a setting where they have been shown to outperform deep learning approaches in multiple machine learning benchmarks13 (12).

It should be noted that direct comparison with prior deep learning studies is complicated by differences in experimental setup, including train–test split strategies, the number of diagnostic classes, and evaluation metrics. Nevertheless, the strong performance of our approach using pre-extracted features suggests that feature engineering remains a viable and potentially superior strategy for focused ECG diagnostic tasks, particularly when interpretability is a priority.

### 5.2 Comparison with imaging-based LVH detection approaches

Establishing LVH in clinical practice rests primarily on cardiac imaging. Cardiac magnetic resonance imaging (CMR) is widely regarded as the anatomical gold standard for LV mass quantification owing to its high spatial resolution and reproducibility, while two-dimensional and three-dimensional echocardiography remain the principal modalities for routine clinical assessment because of their wide availability, portability, and absence of ionising radiation. Chest radiography, although insensitive for early LVH, is the most accessible cross-sectional cardiac imaging modality and has attracted growing interest for opportunistic LVH and cardiomegaly screening when augmented by deep learning.

Recent deep-learning studies on imaging modalities provide an informative context for our ECG-feature-based results. Echocardiography-based approaches trained on large institutional cohorts have reported AUC values in the approximate range of 0.80–0.90 for the detection or precision phenotyping of LVH and related hypertrophic conditions, depending on the operational definition (e.g., LV mass index threshold vs. aetiology-specific phenotyping). CMR-based deep-learning analyses, when applied to cohorts in which CMR-derived LV mass index serves as the reference standard, typically report higher AUC values, reflecting both the gold-standard nature of the modality and the more selected populations involved. Chest-radiography-based deep-learning approaches, although reporting somewhat lower AUC values for LVH *per se*, remain attractive for population-scale screening because of the extraordinary accessibility of the modality.

Set against these benchmarks, the AUC values we observed for interpretable ECG-feature-based models (best AUC 0.9859 on held-out folds) are comparable to or exceed several reported imaging-based benchmarks. Two caveats are essential. First, our reference standard is the diagnostic LVH label adjudicated within PTB-XL/PTB-XL+ rather than imaging-derived LV mass, so our comparison with imaging-based studies should be read as a contextual reference rather than a head-to-head modality-vs.-modality benchmark. Second, the imaging modalities are inherently better suited to quantitative LV mass assessment, while ECG-based approaches detect the electrophysiological footprint of hypertrophy and may therefore exhibit modality-specific failure modes (for example, reduced sensitivity in patients with concentric remodelling without overt voltage criteria, or in patients with poor electrical coupling).

Taken together, ECG-feature-based detection and imaging-based detection are best regarded as complementary rather than competing. ECG offers a low-cost, broadly accessible, rapidly acquired, and operator-independent modality ideally suited to high-throughput first-line screening—for example, in primary care, emergency departments, intensive care units, pre-operative clearance, and population-level cardiovascular screening—while imaging provides the anatomically grounded confirmation necessary for definitive diagnosis and management. The interpretable framework presented here is therefore intended to function as a transparent screening layer that can identify patients with a high probability of LVH for downstream imaging confirmation, rather than as a substitute for imaging-based assessment.

### 5.3 Cross-source comparison of ECG feature extractors

A key contribution of this work is the systematic cross-source comparison of three ECG feature extractors with distinct algorithmic foundations. The marginal performance differences between 12SL, UNIG, and ECGDELI (XGBoost AUC: 0.9859, 0.9853, and 0.9828, respectively) suggest that the underlying electrocardiographic manifestations of LVH are robustly captured by all three algorithms, despite their fundamentally different waveform delineation approaches. This convergence provides reassurance that LVH-related ECG changes are sufficiently pronounced and consistent to be detected regardless of the specific computational methodology used for feature extraction.

The slight performance advantage of commercial algorithms (12SL, UNIG) over the open-source ECGDeli toolkit was most pronounced with Logistic Regression (AUC: 0.9739 and 0.9752 vs. 0.9516), suggesting that the richer feature vocabularies of commercial systems (782 and 702 vs. 529 features) provide greater redundancy that benefits simpler models with limited capacity to learn complex feature interactions. For tree-based models capable of capturing non-linear relationships, this advantage narrowed considerably (*Δ*AUC < 0.003 between 12SL and ECGDELI XGBoost).

The competitive performance of ECGDeli, despite being an open-source tool with a more limited feature set, is encouraging for the broader research community. It demonstrates that interpretable LVH detection with AUC > 0.98 is achievable without reliance on proprietary commercial software, facilitating reproducible research and potential deployment in resource-limited settings where commercial ECG analysis software may not be available.

### 5.4 Clinical interpretation of SHAP feature attributions

The SHAP interpretability analysis provides clinically meaningful insights that validate our models' decision-making processes and align with established cardiology knowledge. The most notable finding across all three feature sources is the predominance of T-wave and ST-segment parameters in the lateral leads (V5–V6), which corresponds to the well-characterized LVH strain pattern. This pattern, characterized by ST depression and asymmetric T-wave inversion in leads V5–V6, reflects subendocardial ischemia and altered repolarization sequences in the hypertrophied myocardium (13, 14).

For the 12SL feature set, the prominence of T + _Dur_V6 (T-wave duration) as the top predictor is noteworthy. While voltage criteria have traditionally dominated ECG-based LVH diagnosis, our findings suggest that temporal aspects of T-wave morphology—specifically, prolonged T-wave duration in lateral leads—carry substantial diagnostic value. This observation aligns with recent electrophysiological studies demonstrating that LVH causes heterogeneous prolongation of repolarization, which manifests as altered T-wave duration and morphology (15).

The importance of R-wave amplitudes in V1 and V5 in the ECGDELI feature set directly validates the Sokolow–Lyon criteria (SV1 + RV5/RV6 > 3.5 mV) (4) and the Cornell voltage criteria (RaVL + SV3 > 2.8/2.0 mV for males/females) (5), which remain the most widely used clinical ECG criteria for LVH. However, the relatively lower ranking of pure voltage features compared to repolarization features in the 12SL and UNIG analyses suggests that repolarization abnormalities may be more discriminative than voltage changes alone, consistent with the known limitation of voltage criteria in isolation (3, 5).

The contribution of T_Amp_aVR as the top feature in the ECGDELI analysis deserves special attention. Lead aVR is often overlooked in routine ECG interpretation, yet it provides a unique perspective as the only standard lead that directly faces the right upper portion of the heart. T-wave changes in aVR reflect reciprocal changes to the lateral leads and may capture global repolarization abnormalities associated with LVH. Recent studies have highlighted the diagnostic utility of aVR in various cardiac conditions (16), and our findings suggest its potential value in automated LVH detection systems.

### 5.5 Role of age and demographic factors

Age consistently ranked among the top five predictors across all three feature sources, with SHAP values of 0.289 (12SL), 0.314 (UNIG), and 0.323 (ECGDELI). This finding reflects the well-established epidemiological association between advancing age and LVH prevalence, whichincreases from approximately 8% in adults under 50 to 15%–20% in those over 65 years [see also Cuspidi et al. (20)] (1). The inclusion of age as a feature effectively captures this prior probability, improving discriminative performance. However, the substantial contribution of ECG features independent of age confirms that the models are not merely leveraging age as a proxy but are extracting genuine electrocardiographic signal.

The significant demographic differences between LVH and normal groups (Table 1)—including older age, male predominance, and different body habitus—are consistent with known risk factors for LVH, including hypertension, which increases with age and disproportionately affects men (2). The lower body weight observed in the LVH group may appear counterintuitive given the association between obesity and LVH, but likely reflects the older age and potential comorbidities (including heart failure-related cachexia) prevalent in this group.

### 5.6 Practical implications for model selection

The narrow performance range among tree-based models (*Δ*AUC < 0.004 between XGBoost and Random Forest within each feature source) suggests that the choice of specific tree-based algorithm is less critical than the choice of feature source and the decision to use ensemble methods over linear models. This finding has practical implications for clinical deployment: institutions may select models based on computational efficiency, regulatory considerations, or software compatibility without substantially compromising diagnostic performance.

The notable performance drop observed with Logistic Regression, particularly for ECGDELI features (AUC = 0.9516 vs. 0.9828 for XGBoost), underscores the importance of non-linear modeling in this domain. We further note that our best AUC of 0.986 is comparable to deep-learning approaches reported on similar ECG analysis tasks. We do not claim superiority over deep learning models; rather, we observe that when commercial-grade ECG features are available, traditional interpretable models can serve as a competitive and clinically transparent alternative. We view feature-based and end-to-end deep-learning approaches as complementary, with the trade-off between performance and interpretability depending on the specific clinical context. This complementarity, rather than a binary choice, is a central message of our study. ECG features exhibit complex interactions (e.g., the significance of R-wave voltage depends on patient age and QRS duration), which linear models cannot fully capture. The SHAP dependence plots (Figure 5) visually confirm these non-linear relationships, with threshold effects and interaction patterns that explain the superiority of tree-based methods.

From a clinical deployment perspective, the precision–recall analysis (Figure 2D) is particularly relevant. All three feature sources achieved average precision > 0.95, indicating reliable positive predictive performance even in the presence of class imbalance (19.3% LVH prevalence). The high precision (0.9242 for the best model) suggests that positive predictions would have a low false alarm rate, which is essential for maintaining clinician trust in automated screening systems. The recall of 0.8778, while lower, ensures that the majority of LVH cases are correctly identified, with the model demonstrating a clinically acceptable sensitivity–specificity trade-off.

### 5.7 Value of interpretability for clinical integration

A central motivation of this study was to develop models whose predictions can be meaningfully explained to clinicians. The SHAP framework provides several advantages in this regard. Global feature importance rankings (Figure 4) enable clinicians to verify that model logic aligns with domain knowledge, building trust in the system. Local SHAP explanations (available for individual patients) can identify which specific ECG abnormalities contributed to a given patient's LVH prediction, supporting clinical decision-making and potentially guiding targeted follow-up investigations.

The alignment of our model's feature attributions with established electrocardiographic knowledge is reassuring. A model that identifies T-wave strain patterns and R-wave voltage criteria as primary decision factors is applying logic that cardiologists can immediately recognize and evaluate. This stands in contrast to deep learning approaches where model decisions, even when correct, remain opaque to clinical scrutiny. As Rudin (8) has argued, interpretable models should be preferred over *post-hoc* explanations of black-box models in high-stakes medical decisions, and our results demonstrate that interpretability need not come at the cost of predictive performance.

### 5.8 Limitations

Several limitations should be acknowledged. First, the PTB-XL dataset originates from a single institutional source (Schiller AG, Germany), and the generalizability of our findings to diverse populations with different ethnic compositions, body habitus distributions, and disease prevalences requires external validation. ECG-based LVH criteria are known to have variable performance across racial and ethnic groups (17), and the predominantly European composition of PTB-XL may limit applicability to other populations.

Second, the LVH labels in PTB-XL are ECG-based diagnostic annotations assigned by cardiologists, rather than echocardiographic confirmations of increased left ventricular mass. This introduces potential label noise, as ECG-diagnosed LVH does not perfectly correspond to anatomical LVH. Studies have shown that the sensitivity of ECG criteria for echocardiographic LVH varies widely (3–5), meaning that some true LVH cases may be labeled as normal, and some normal cases may carry false LVH labels. Future studies should incorporate echocardiographic or cardiac MRI ground truth for more rigorous validation.

Third, our binary classification framework does not address the spectrum of LVH severity or distinguish between concentric and eccentric hypertrophy patterns, which have different etiologies, clinical implications, and prognoses. A multi-class or regression approach incorporating LV mass index could provide more clinically nuanced outputs.

Fourth, the pre-extracted features, while comprehensive, may not capture all relevant ECG information available in raw waveforms. Subtle morphological patterns (e.g., QRS fragmentation, late potentials) that are not explicitly quantified by the feature extraction algorithms may be missed. Conversely, raw-signal deep learning approaches may detect patterns that are not represented in traditional ECG measurements.

Fifth, height and weight data were missing for a substantial proportion of the cohort (65.3% and 47.9%, respectively), limiting our ability to calculate body surface area-adjusted metrics or to fully characterize the influence of body habitus on model predictions. Additionally, clinical information such as blood pressure, medication use, and echocardiographic measurements were not available, precluding a comprehensive clinical context analysis.

Sixth, the class imbalance (19.3% LVH) may affect model calibration despite mitigation through class weighting. While we employed appropriate evaluation metrics (F1, precision–recall curves) that account for imbalance, the calibration of predicted probabilities was not formally assessed. Finally, hyperparameter optimization was not performed via exhaustive search, and additional performance gains may be achievable through systematic tuning.

Seventh, the PTB-XL+ dataset does not include paired cardiac imaging (echocardiography, CMR, or chest radiography) for the same patients, and therefore does not permit a direct, patient-level comparison between our ECG-feature-based predictions and imaging-derived LVH assessment. The comparison with imaging-based deep-learning benchmarks presented in Section 4.2 is consequently contextual rather than head-to-head. Prospective multi-modal cohorts that link 12-lead ECG to echocardiography or CMR acquisitions on the same patients constitute the appropriate setting for such validation and represent a clear direction for future work.

### 5.9 Future directions

Several promising directions emerge from this work. First, the integration of multi-source features—combining 12SL, UNIG, and ECGDELI features into a unified feature matrix—could potentially leverage complementary information from different extraction algorithms and improve performance further. Feature selection or dimensionality reduction techniques would be essential to manage the resulting high-dimensional feature space.

Second, hybrid approaches that combine pre-extracted features with raw-signal deep learning representations warrant investigation. Such models could exploit the interpretability of expert-defined features while capturing additional patterns detectable only from raw waveforms. Knowledge distillation from deep learning models into interpretable feature-based models represents another avenue for combining the strengths of both paradigms.

Third, prospective clinical validation in real-world ECG interpretation workflows is essential before deployment. This should include assessment of calibration, decision threshold optimization for specific clinical use cases (screening vs. confirmatory), and evaluation of the impact of SHAP explanations on clinician decision-making through user studies. Multi-center validation across diverse patient populations would further establish generalizability.

Fourth, the development of patient-specific risk scores incorporating longitudinal ECG feature trajectories represents a promising direction. Serial ECG monitoring with interpretable ML could enable early detection of LVH progression, potentially allowing timely therapeutic intervention before the development of clinically significant hypertrophy.

## 6 Conclusion

This study demonstrates that interpretable machine learning models using commercial ECG features from the PTB-XL+ dataset achieve excellent performance for LVH detection, with AUC values exceeding 0.98. SHAP analysis provides clinically transparent feature attributions that align with established electrophysiological knowledge of LVH, including the predominance of T-wave changes in lateral leads, R-wave voltage criteria, and age-related risk. The comparable performance of commercial (12SL, UNIG) and open-source (ECGDELI) feature extractors supports the broader applicability of this approach. By combining high diagnostic accuracy with meaningful interpretability, this methodology offers a practical pathway for integrating automated LVH screening into routine clinical ECG interpretation workflows.

## Data Availability

The original contributions presented in the study are included in the article/Supplementary Material, further inquiries can be directed to the corresponding author/s.
